# Low-power biomimetic ionic thermoelectric device for multi-gas olfaction

**DOI:** 10.1093/nsr/nwag320

**Published:** 2026-05-28

**Authors:** Gongze Liu, Cheng Chi, Jiacheng Ji, Keqiao Li, Meng Li, Yucheng Ding, Weiqi Zhang, Wenying Tang, Zhiyong Fan, Baoling Huang

**Affiliations:** Department of Mechanical and Aerospace Engineering, The Hong Kong University of Science and Technology, Hong Kong 999077, China; Key Laboratory of Power Station Energy Transfer Conversion and System of Ministry of Education, School of Energy Power and Mechanical Engineering, North China Electric Power University, Beijing 110026, China; School of Physics, Engineering and Computer Science, University of Hertfordshire, Hatfield AL10 9AB, UK; Department of Mechanical and Aerospace Engineering, The Hong Kong University of Science and Technology, Hong Kong 999077, China; Department of Mechanical and Aerospace Engineering, The Hong Kong University of Science and Technology, Hong Kong 999077, China; Department of Mechanical and Aerospace Engineering, The Hong Kong University of Science and Technology, Hong Kong 999077, China; Department of Electronic and Computer Engineering, The Hong Kong University of Science and Technology, Hong Kong 999077, China; Department of Electronic and Computer Engineering, The Hong Kong University of Science and Technology, Hong Kong 999077, China; Department of Electronic and Computer Engineering, The Hong Kong University of Science and Technology, Hong Kong 999077, China; Department of Electronic and Computer Engineering, The Hong Kong University of Science and Technology, Hong Kong 999077, China; Department of Mechanical and Aerospace Engineering, The Hong Kong University of Science and Technology, Hong Kong 999077, China; HKUST Shenzhen-Hong Kong Collaborative Innovation Research Institute, Shenzhen 518000, China; Thrust of Sustainable Energy and Environment, The Hong Kong University of Science and Technology, Guangzhou 511400, China

**Keywords:** ionic thermoelectrics, biomimetic olfactory device, solid-state ionic polymer, multi-gas sensing, low-power infrared detection

## Abstract

The escalating demand for intelligent multi-gas sensing has driven the need for high-performance artificial olfactory systems with enhanced power efficiency. This work proposes an innovative gas sensing mechanism enabled by the giant ionic-thermoelectric effect in solid-state ionic polymer, which overcomes the low thermopower-to-thermal conductivity (*S/κ*) limitation of the conventional thermoelectric materials. Using wafer-scale microfabrication technology, we demonstrate the first functional ionic-thermoelectric biomimetic olfactory device that can simultaneously resolve mixed gas analytes. This monolithic platform integrates multiple sub-100 μm sensing units, each combining an ionic-thermoelectric module with a narrow-bandpass optical filter, to selectively resolve the contributions of individual gas species in mixed-gas environments. The device achieves a record responsivity of 2340 V/W, a 20-fold improvement over commercial detectors (typically <200 V/W) and limits of detection of 1.42 ppm, 0.15 ppm, and 1.16 ppb for CO_2_, CH_4_ and CO, respectively. This work establishes a promising route to next-generation artificial olfactory systems based on the ionic-thermoelectric effects.

## Teaser

A miniaturized artificial olfactory device uses ionic thermoelectric materials to detect multiple gases with high sensitivity and low power consumption.

## INTRODUCTION

The global demand for intelligent gas sensing systems is rapidly escalating, driven by critical applications in environmental monitoring, healthcare diagnostics, food safety, and industrial automation [1–5]. Inspired by the human olfactory system, which can distinguish complex odor profiles with high sensitivity and selectivity, electronic noses (e-noses) are engineered to replicate and even surpass these capabilities through sensor arrays and pattern recognition algorithms [6–8]. Among the various gas sensing technologies adopted in modern e-nose, such as electrochemical sensors, nondispersive infrared (NDIR) sensors, photoionization sensors and metal oxide sensors [7,9–14], NDIR sensors are particularly attractive due to their unique merits such as high reliability, long lifetime, excellent selectivity, and low operation temperature [7,9–14]. The modern NDIR sensors directly detect infrared absorption fingerprints of gases caused by characteristic molecular vibrations and are very suitable for long-term continuous sensing of heteroatomic gases such as hydrocarbons, NO*_x_*, CO, and CO_2_. As the key component of NDIR gas sensors, the infrared detectors directly determine the performance and cost of the gas sensing system. Thermoelectric (TE) infrared sensors based on the ‘Seebeck effect’, in which a temperature difference can induce a thermal voltage due to the thermal diffusion of charge carriers in solids, are among the most common infrared detectors used for gas sensing, owing to their ultralow energy consumption, mature fabrication process, and high stability [15]. They are especially suitable for long-term continuous operation due to their passive nature, showing advantages over other infrared sensing technologies such as photovoltaics [16] and bolometers [17,18].

However, state-of-the-art TE infrared sensors often suffer from relatively low responsivity, typically around 100 V/W, and from a low response voltage [19], primarily due to the unsatisfactory performance of TE materials, particularly their low thermopower (*S*) to thermal conductivity (*κ*) ratio [19]. This ratio is a key parameter governing thermoelectric sensor performance, because *κ* determines heat dissipation and thus the achievable temperature rise under incident radiation, while *S* governs the conversion of this temperature difference into electrical voltage. The responsivity (*R*) of a TE infrared sensor can be expressed as [20]: $R = \frac{{4\eta \delta S}}{{\kappa \pi {d}^2}}$, where *η, δ* and *d* are the absorption efficiency, thickness of the sensing layer and characteristic diameter of the detector, respectively [20]. This relationship explicitly shows that *R* is proportional to the *S*/*κ* ratio. Therefore, a higher thermopower enhances the signal generation, while a lower thermal conductivity suppresses heat dissipation, both contributing to improved responsivity. However, the conventional electronic-based TE materials, such as doped semiconductors (e.g. Bi_2_Te_3_, PbTe), face an intrinsic trade-off. Reducing the *κ* via heavy doping or alloying increases carrier-phonon scattering, inadvertently reducing *S* and degrading overall performance. For instance, n–type Bi_2_Te_2·7_Se_0.3_ achieves a decent *S* of *−*230 μV/K but simultaneously suffers from high *κ* = 1.5 W/(m·K), yielding a low absolute *S*/*κ* ratio of 153 μV·m/W, which is insufficient for high-sensitivity applications [21,22]. This physical *S*/*κ* bottleneck not only restricts responsivity but also imposes low specific detectivity and large device size. Such limitations become particularly vital in low-power high-performance gas sensing with miniature device size, where the ability to generate a significant output voltage from a weak narrowband infrared signal is crucial.

Recent advances in ionic-thermoelectric (*i*-TE) materials offer a promising solution to the *S*/*κ* dilemma. Unlike electronic TE materials, *i*-TE systems employ ion migration under temperature gradients, akin to the ion flux mechanisms underlying neural signaling in biological olfaction, to generate substantial thermoelectric voltages [23–27]. This decoupling of *S* and *κ* arises from the distinct transport mechanisms of ions and phonons, bypassing the electronic material’s inherent trade-offs. The Soret effect enables *i*-TE composites to achieve giant thermopower values exceeding tens of millivolts per kelvin, which are several orders of magnitude higher than conventional TE materials, while maintaining ultralow thermal conductivity of <0.5 W/(m·K) [26,28–30]. This unique combination makes *i*-TE materials ideal for self-powered, ultrasensitive infrared (IR) sensing.

Moreover, the wafer-scale microfabrication offers several critical advantages for practical *i*-TE sensing systems, including reproducible batch fabrication, improved device-to-device uniformity, and compatibility with dense array integration. These features are especially important for biomimetic olfactory devices, where consistent performance across multiple sensing units is required for reliable pattern recognition. In addition, wafer-scale processing supports sub-100 μm miniaturization and is readily compatible with standard chip-level fabrication, optical filter integration, and packaging strategies, thereby providing a practical route toward large-scale manufacturing and deployment of integrated *i*-TE sensing platforms. However, current *i*-TE systems predominantly utilize liquid or gel electrolytes [31,32], suffering from mechanical instability, solvent evaporation, and incompatibility with standard microfabrication processes. Addressing the existing material and fabrication challenges is critical to realizing the full potential of *i*-TE technology for scalable and high–performance odor analysis.

In this work, we demonstrate the first fully integrated biomimetic olfactory device (BOD) with multi-gas detection capability, which is constructed from a solid-state *i*-TE array fabricated via wafer-scale microfabrication technology. This design leverages solvent-free *i*-TE polymers, which exhibit giant ionic thermopower and ultralow thermal conductivity, leading to exceptional *S/κ* ratios (>24 mV·m/W). The proposed BOD integrates multiple sub-100 μm sensing units with fast response and excellent selectivity, each consisting of a miniature *i*-TE detector and a narrow-band pass optical filter, to form a self-powered platform capable of simultaneously detecting multiple gas species including CO_2_, CH_4_ and CO as representative demonstrations. The BOD sensing units demonstrate an ultrahigh responsivity of 2340 V/W, almost 20 times higher than commercial TE detectors. The calculated limits of detection (LOD) are 1.42 parts per million (ppm) for CO_2_, 0.15 ppm for CH_4_, and 1.165 parts per billion (ppb) for CO, indicating superior performance compared with commercial NDIR gas sensors, which typically operate at ppm level [9,33]. Moreover, the sensing architecture is inherently extensible to a broader range of gas analytes through appropriate filter design and array configuration. Overall, this proposed BOD offers a unique combination of low-power operation, high sensitivity, and multi-gas detection capability, showing the great potential of implementing the biomimetic ionic detection mechanism in advanced gas sensing devices.

## RESULTS

**Figure 1.**
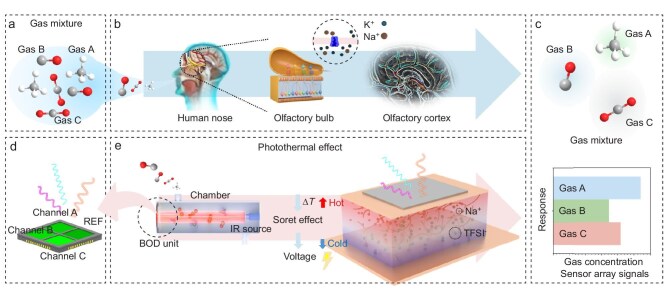
Comparison of the human olfactory system and biomimetic olfactory devices. (a) Gas molecules in a mixture. (b) Schematic of gas detection in human olfactory bulbs and signal generation in olfactory cortex. (c) Illustration of the recognition results from humans and BOD. (d) Schematic of BOD structure. (e) Schematic of NDIR module and BOD units together with the signal generation principle in an ionic-thermoelectric (*i*-TE) infrared sensor.

The human sense of smell originates from neural signals generated by the interaction between olfactory receptors and gas molecules, which are then processed by complex neural networks in the brain. When gas molecules enter the nasal cavity and interact with the olfactory bulb (Fig. 1a and b), they stimulate olfactory receptor cells to generate an action potential through sodium-potassium pumps which regulate the concentration gradients of Na⁺ and K⁺ ions across cell membranes. These electrical signals are transmitted via the olfactory nerve to the olfactory cortex, where they are decoded into specific odor information (Fig. 1c). Inspired by this neural response, we developed an innovative strategy employing *i*-TE polymer composites to construct a biomimetic olfactory device as illustrated in Fig. 1d. Specifically, the proposed device mimics the array-based sensing feature of human olfaction and, more importantly, its ion-involved electrical signaling concept, while its physical transduction mechanism is based on infrared-modulated photothermal conversion and ionic thermoelectric response. Moreover, it consists of an infrared light source (EMIRS200), a gas chamber allowing selective infrared absorption, and an ionic olfactory detection unit that integrates an absorption layer with an *i*-TE module supported by an SU-8 mold. The working principle relies on the combination of photothermal and Soret effects. As shown in Fig. 1d, the infrared light emitted by the broadband infrared source will experience highly selective absorption caused by the resonance of gas molecule vibrations in the gas chamber, and the intensity attenuation at those characteristic wavelengths depends on the optical path and gas concentrations. The infrared light modulated by different gas species then passes through the narrowband filters and gets absorbed by the absorption layer, creating a temperature gradient that triggers the Soret effect in the *i*-TE polymer composite. The anions and cations in the polymer will thermally diffuse at different rates and their asymmetric Eastman entropies of transfer generate a measurable voltage signal, enabling precise quantification of light intensity variation within selected bands and in turn target gas concentrations through voltage monitoring (Fig. 1e). The *i*-TE polymer composite can achieve a much higher *S/κ* than conventional TE materials and makes it an ideal candidate for thermal sensing. Furthermore, the fabrication process for *i*-TE polymers is both cost-effective and environmentally friendly compared to traditional inorganic TE materials.

**Figure 2.**
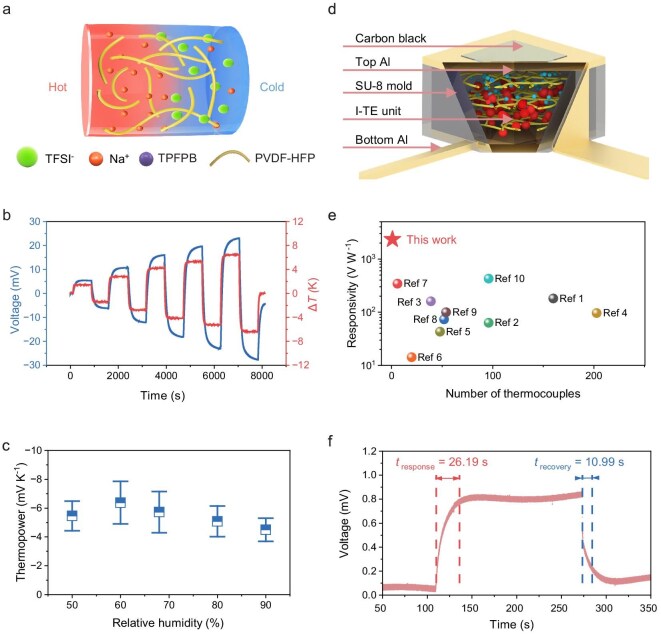
Thermoelectric performance of *i*-TE materials and sensing units. (a) Diagrammatic illustration of the n-type *i*-TE material used. (b) Measured ∆*V/*∆*T* curves of the PVDF-HFP/NaTFSI/TPFPB/PC, and (c) its thermopower at different relative humidity. (d) Schematic of a BOD sensing unit. (e) Responsivity comparison between existing TE thermopiles and the *i*-TE BOD sensing unit [35–44]. (f) Thermal response of a BOD sensing unit with respect to time.

Herein, an all-solid-state *i*-TE polymer is integrated into the biomimetic olfactory device. This *i*-TE polymer comprises poly(vinylidene fluoride-co-hexafluoropropylene) (PVDF-HFP) matrix, sodium bis(trifluoromethylsulfonyl)imide (NaTFSI) salt and propylene carbonate (PC) plasticizer (Fig. 2a) and the corresponding chemical structures of the molecules are shown in Fig. S1. Specifically, PVDF-HFP provides a porous ion-conducting polymer matrix with low thermal conductivity and good film-forming ability, which is favorable for maintaining a temperature gradient and device integration. The NaTFSI serves as the ionic source, while PC acts as a plasticizing medium to promote ion dissociation and transport, enabling a relatively high ionic thermopower. Low-molecular-weight tris(pentafluorophenyl)borane (TPFPB) is further introduced to tune the ion-transport asymmetry through selective capture with Na^+^ ions, which suppresses cation thermodiffusion and shifts the thermoelectric response toward n-type behavior. This design is beneficial for obtaining a stable n-type *i*-TE polymer suitable for reliable sensing operation under different humidity conditions [34]. To measure ionic thermopower accurately, an in-plane setup with meticulous calibration has been constructed (Fig. S2). According to prior research, the hot and cool sides of the samples are electrically connected to the positive and negative terminals of a voltmeter [34]. Figure 2b shows the voltage ∆*V* generated under a temperature difference ∆*T*, illustrating typical n-type behaviors. The ionic thermopower (*S_i_*) can be determined by fitting the slope of ∆*V/*∆*T* according to the equation below:


(1) \begin{eqnarray*}
{S}_i = \ - \frac{{V( {{T}_{\rm H}} ) - V( {{T}_{\rm C}})}}{{{T}_{\rm H} - {T}_{\rm C}}},
\end{eqnarray*}

where *V* (*T*_H_) and *V* (*T*_C_) are the potentials corresponding to the high (*T*_H_) and low temperatures (*T*_C_), respectively. Figure 2c shows the thermopower of the n-type PVDF-HFP/NaTFSI/TPFPB/PC *i*-TE polymer composite under different relative humidities. This n-type polymer composite demonstrates a weak humidity dependence and stable performance with thermopower ranging from ∼*−*5 to *−*6 mV/K within the testing humidity, which is important for the reliability of the sensing unit under different application scenarios. At high humidity, absorbed water can promote NaTFSI dissociation and release more mobile free Na^+^ ions that are otherwise partially associated with TPFPB. This increased cation contribution may partially offset the anion-dominated thermodiffusion, leading to a slight decrease in the magnitude of the negative thermopower. Figure 2d illustrates the BOD sensing unit, which comprises an absorption layer made of carbon black, a solid *i*-TE polymer composite contained by a SU-8 mold, and two Al electrodes on the top and bottom of the mold, in which the carbon black was selected for its broadband infrared absorption, efficient photothermal conversion, low cost, and compatibility with the solid-state *i*-TE device, enabling localized heating for voltage generation. After packaging, the BOD sensing unit with the n-type *i*-TE polymer composite demonstrates stable performance under humidity ranging from 30% to 90% (detailed data are shown in Figs S3–S5). Moreover, the long-term stability of the packaged BOD device was monitored under ambient conditions for nearly two months, as shown in Fig. S6. It recorded the generated voltage of the device under the same infrared source to compare the stability of the fabricated BOD device. Clearly, the output voltage difference remained stable at approximately 1.0–1.2 mV throughout the test period of over 45 days, without any obvious downward trend or degradation in signal response. The repeated transient response measurements also remained highly consistent, further confirming the operational stability of the packaged device. Thus, the adoption of the relatively humidity-insensitive n-type *i*-TE polymer and device-level insulating packaging ensures good device stability under different humidity environments during long-term operation.

Figure 2e shows the comparison of the responsivity of the attained *i*-TE sensing unit and those of thermopile sensors reported in the literature [35–44]. Owing to the relatively low performance of conventional electronic TE materials, commercial thermopile sensors often leverage microfabrication technology to integrate hundreds of thermocouples in series, thus boosting the sensor responsivities to a typical value of ∼200 V/W. In contrast, the *i*-TE polymer used in this work has an intrinsically high *S* of ∼**–**6 mV/K and a low *κ* of ∼0.25 W/(m·K), which is beneficial for maintaining a remarkable temperature difference during the measurement and in turn a high responsivity of the device. These merits together with the excellent thermal stability mentioned above make PVDF-HFP-based *i*-TE polymer an excellent candidate for ionic thermoelectric applications. Interestingly, the *i*-TE polymer used in this work can achieve an ultrahigh *S/κ* ratio of 24 mV·m/W, enabling a giant responsivity of 2340 V/W with just a single *i*-TE sensing unit. This ionic design greatly reduces fabrication complexity while significantly enhancing sensor voltage output. Figure 2f and Fig. S7 further show the thermal response of the BOD sensing unit, exhibiting a response time of 26.19 s and a recovery time of 10.99 s, comparable to those of commercial gas sensors (typically around 30 s). The shorter recovery time compared with the response time is likely attributed to the asymmetric heating and cooling processes. During the response stage, heat must accumulate in the device to establish a sufficient temperature gradient and corresponding ionic thermoelectric output. In contrast, during recovery, the temperature gradient decays more rapidly because the bottom substrate and ceramic package serve as an efficient heat sink (cold side), which accelerates heat dissipation from the top *i*-TE material. As a result, the output signal returns to the baseline faster than it rises during heating.

**Figure 3.**
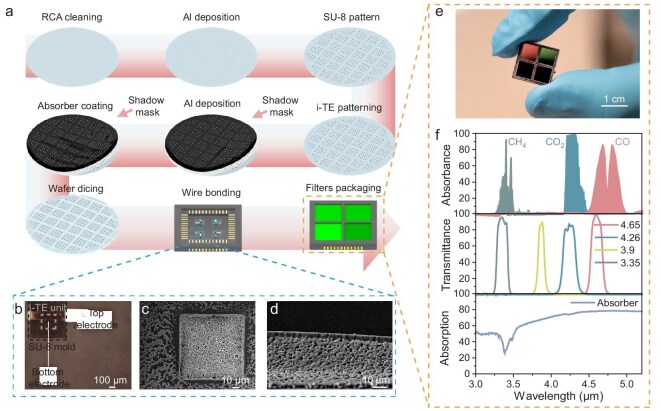
The schematic and characterization of BOD sensing units. (a) Process flow of BOD sensing units. (b) Image of the BOD sensing unit. (c) Top view SEM image of *i*-TE unit. (d) SEM image of the *i*-TE unit cross-section. (e) The image of BOD with the CLCC package and filters. (f) Absorption spectra of the target gases (top), measured transmittance spectra of the narrow bandpass filters (middle), and measured absorption spectra of the absorber (bottom).

Further, the fabrication process for the BOD sensing units, which are fabricated on 4-inch quartz wafers, is shown in Fig. 3a. The 4-inch quartz wafers with a thickness of 500 µm were subjected to a standard Radio Corporation of America (RCA) cleaning procedure to remove organic and inorganic contaminants. Thereafter, a 300 nm-thick aluminum layer was deposited through a shadow mask via physical vapor deposition to form the bottom electrode. Subsequently, a patterned SU-8 photoresist mold was formed atop the bottom electrode using standard photolithography techniques. The *i*-TE polymer solution was then dispensed into the SU-8 mold cavities and baked in a vacuum oven to form solid-state *i*-TE pillars. To form the top electrode, a 300 nm-thick layer of aluminum is deposited on the wafers using a sputtering process with a shadow mask. Alignment of the shadow mask under a microscope ensures comprehensive coverage of the *i*-TE pillars and electrodes. A layer of 2-μm black paint is spray-coated on the wafers with a shadow mask to form absorbers. The completed wafers were then singulated using a laser wafer dicing system, and individual dies were wire bonded onto ceramic leadless chip carrier-48 (CLCC-48) packages. Finally, narrow-bandpass filters are implemented to define multiple sensing channels.

A typical BOD sensing unit is shown in Fig. 3b, which consists of a top and a bottom electrode, an SU-8 mold, and an *i*-TE unit. Detailed scanning electron microscopy (SEM) top view and cross-sectional views of a sensing unit are presented in Fig. 3c and d, respectively, showing the *i*-TE sensing unit has a size of ∼100 μm and a thickness of ∼35 μm. It reveals that the filled polymer layer possesses a relatively uniform thickness across the cavity and maintains good contact with the cavity walls, and there are no obvious discontinuous regions, or severe overflow outside the designed cavity are observed, confirming successful filling of the cavity. Figure 3e depicts the BOD module featuring a CLCC-48 package and four narrow-bandpass filters (Fig. S8) corresponding to four sensing units. Specifically, each sensing unit is equipped with a narrow-band optical filter that isolates a distinct infrared wavelength region. Because different gases exhibit characteristic absorption features at different wavelengths, integrating multiple units into one module enables multi-channel spectral discrimination, allowing the system to distinguish target gases more reliably and reducing cross-interference. In this way, the module provides not only stronger functionality than a single unit but also improved selectivity and identification capability through comparative analysis of the outputs from different channels. In this work, we selected three different gases including CO_2_, CO and CH_4_ for demonstration, which are common gas species raising safety concerns in the indoor environment. Figure 3f displays the IR absorption spectra of the target gases at the top, where CO_2_, CO, and CH_4_ molecules exhibit strong characteristic absorption peaks at wavelengths of 4260 nm, 4650 nm and 3350 nm, respectively. The absence of overlap in this range makes it an ideal choice for detecting these gases with high selectivity based on their absorption properties. The measured transmittance spectra of the narrow bandpass filters, shown in the middle, demonstrate transmittance levels exceeding 80% at the corresponding IR absorption peaks of the target gases, with no overlap between them. Moreover, the 3900 nm channel is introduced as a reference channel, since it does not correspond to any characteristic infrared absorption peak of the target gases. As indicated by the yellow transmission peak in Fig. 3f, the corresponding multilayer-coated filter exhibits high transmittance around 3900 nm while blocking other wavelength regions. Therefore, this channel mainly reflects the baseline intensity of the infrared source and possible optical-path attenuation, enabling compensation for source instability during long-term operation and improving the reliability of gas recognition. At the bottom of Fig. 3f, the measured absorption spectra of the absorber are also displayed. To be noticed, CO_2_, CH_4_, and CO are selected as representative target gases, while the underlying sensing strategy is general and not limited to these specific analytes. In addition, the filter bandwidth is an important factor affecting gas selectivity, because it determines the extent to which the transmitted spectral window overlaps with the characteristic absorption band of the target gas and with possible interfering species. In general, a narrower bandwidth can suppress spectral cross-interference and improve selectivity, although it may also reduce the transmitted optical power and thus weaken the signal intensity. The bandwidth is also affected by the characteristic peak width of the target gas: a distinctive strong and narrow absorption peak requires a narrow bandwidth, but a wider characteristic absorption peak justifies a relatively wider bandwidth of the filter. Therefore, the filter bandwidth should be carefully optimized to balance selectivity and signal strength according to the absorption spectra of target gases.

### The BOD response to a single-target gas

**Figure 4.**
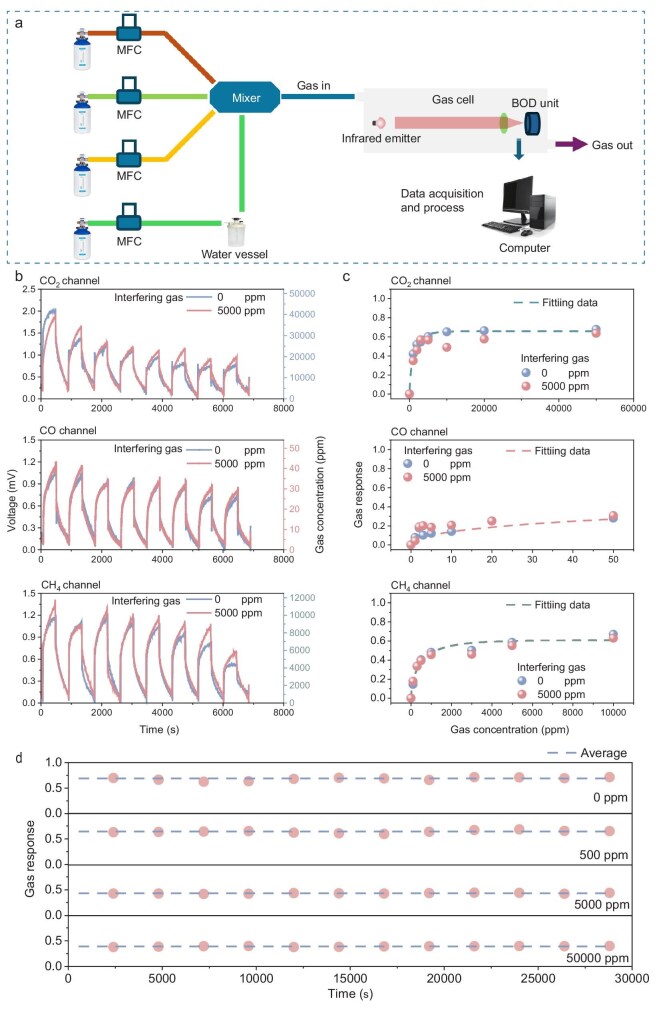
The BOD response to a single-target gas. (a) Schematic of NDIR sensor test platform. (b) Voltage response of BOD with and without interfering gas as a function of target gas concentration. (c) Gas response of BOD as a function of target gas concentration. The blue dots stand for the measured data from pure target gas, the red dots stand for the measured data with interfering gases, and the dashed lines are the fitting curves based on the modified Beer-Lambert law. (d) Repeatability test results of the BOD sensing unit with CO_2_ at different concentrations.

An NDIR gas measurement platform has been established to facilitate the calibration and performance evaluation of the BOD sensing module. As illustrated in Fig. 4a, the system comprises a custom-built gas-sensing setup designed for precise control and measurement. Gas cylinders containing target analytes are connected to mass flow controllers (MFCs), which regulate the flow rates of individual gases. Moreover, the N_2_ stream was subsequently passed through a water vessel to control the relative humidity of the gas mixture. According to our tests, water vapor shows a negligible influence on the measured results. These gases are subsequently directed into a mixing chamber, where they are thoroughly blended to achieve the desired composition. The mixed gas stream is then introduced into a white gas cell housing the sensing module. Inside the gas cell, a commercial infrared emitter (EMIRS200) is employed and controlled via a LabVIEW interface to ensure stable and programmable IR output. The infrared beam passes through the gas cell and is then filtered through a narrow-bandpass optical filter. Subsequently, the infrared signal is converted into a heat signal by the absorption layer and then transformed into a voltage signal by the *i*-TE unit. A computer is integrated into the system to enable automated data acquisition and control of experimental parameters. The complete configuration of the measurement system is detailed in Fig. S9.

To quantitatively assess the performance of the *i*-TE NDIR sensors in detecting target gases, a parameter termed gas response ∆*V/V*_0_ is introduced. *V*_0_ denotes the initial voltage output of the *i*-TE sensors when the chamber is filled with pure nitrogen gas. *V* represents the voltage output of the *i*-TE sensors when the target gases are introduced into the chamber. The change in output voltage caused by the target gas is denoted as ∆*V = V−V*_0_. To further analyze and predict the gas response of the *i*-TE NDIR sensors, a modified Beer-Lambert law is introduced and employed to fit the data, as given by [45–47]:


(2) \begin{eqnarray*}
\Delta V/{V}_0 = \textit{span}*({e}^{ - \varepsilon l{x}^c} - 1).
\end{eqnarray*}

Here the coefficient *span*, ranging from 0 to 1, accounts for the fact that the absorption of infrared radiation cannot reach 100%, owing to the inevitable energy loss during the infrared beam transmission through the gas cell. The coefficient *ε* symbolizes the effective absorption coefficient of the target gas (Fig. S10 and Note S1). *l* denotes the length of the optical path, *x* stands for the concentration of target gases, and *c* is the power factor for fitting with the actual absorption data.

Figure 4b illustrates the responses of the *i*-TE NDIR sensing module to individual target gases in the presence of interfering gases. The concentration of interfering gases was fixed at 5000 ppm for all the measurements. During CO_2_ and CO detections, CH_4_ was introduced as the interfering species, whereas CO_2_ served as the interferent in CH_4_ measurements. The primary vertical axis represents the measured voltage signal, with blue and red lines representing the responses of the target gases with and without the presence of the interfering gases, respectively. A color bar is included to visualize the precise concentration of the target gases. The tested concentrations range from 0–50,000 ppm for CO_2_, 0–50 ppm for CO, and 0–10,000 ppm for CH_4_. The CO concentration was limited to 50 ppm to comply with laboratory safety regulations. Initial peak voltages were recorded under pure nitrogen conditions, where infrared transmission is maximal and the voltage signal reaches its highest value. As the concentration of target gases increases, absorption of infrared radiation at their characteristic wavelengths leads to a reduction in transmitted IR intensity and in turn a decrease in the voltage signal. The close alignment between the blue and red curves across all gas types demonstrates the excellent selectivity of the BOD device. This excellent selectivity is attributed to the fundamental principles of NDIR sensing, wherein each gas exhibits distinct absorption features with minimal spectral overlap.

Moreover, the utilization of ionic thermoelectric materials enables the BOD device to generate millivolt-level signals without the need for external amplification, offering a significant advantage over conventional NDIR sensors that typically require power-consuming amplifiers. The amplitude of the voltage signal varies across each pixel due to differences in absorption and fabrication inconsistencies. The experimental fitting with the modified Beer–Lambert law is presented in Fig. 4c (see Note S1 for more details of the Beer-Lambert law fitting). The majority of the experimental data align closely with the predicted fitting curves, with the coefficient of determination (*R*^2^) exceeding 95%, indicating a strong correlation between the model and observed sensor behavior. The saturation-like response observed at relatively high gas concentrations mainly originates from the Beer-Lambert-type infrared absorption process. At low concentrations, increasing gas concentration leads to a rapid increase in characteristic IR absorption, resulting in a pronounced change in the transmitted IR intensity and thus the sensor output. However, once the characteristic absorption band becomes sufficiently strong, the transmitted IR intensity is already substantially attenuated, so further increases in gas concentration produce only a limited additional change in the voltage response. This leads to the apparent saturation behavior in Fig. 4c. Such behavior indicates high sensitivity in the low-concentration regime, which is beneficial for trace-gas detection, but also suggests a compressed dynamic range at higher concentrations. The detectable range could be further tuned by optimizing the optical path length, selecting suitable absorption bands, or redesigning the narrow-band filter to balance sensitivity and dynamic range. The stability of the gas sensors over a long period is also critical to reliable measurements in practice. As an example, Fig. 4d shows the long-term repeatability test results of the BOD CO_2_ channel at different CO_2_ concentrations. During the test period of ∼30,000 s, the CO_2_ sensing unit demonstrates consistent results over a concentration range of 0 ppm to 50,000 ppm, with fluctuation less than 5%, highlighting the excellent stability and reliability of the BOD device.

### The BOD response to mixed multiple target gases

**Figure 5.**
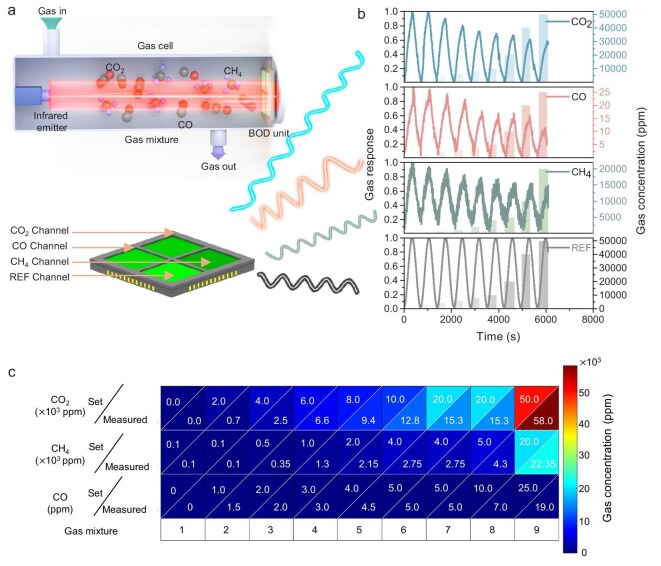
BOD response to mixed target gases. (a) Schematic of the multi-channel gas test platform. (b) Voltage response of BOD as a function of target gas concentration. (c) Gas response of BOD to various gas mixtures with different compositions. The upper left corner of a cell in each column displays the set values, while the lower right corner displays the measured values.

In many applications, the simultaneous detection of multiple gases is highly demanded. By implementing four narrow-bandpass optical filters, the BOD module is capable of detecting infrared signals at four distinct wavelengths, enabling multi-gas detection. The schematic of the multi-channel *i*-TE NDIR gas-sensing platform is presented in Fig. 5a. The chamber is initially purged with nitrogen (N_2_) for 30 minutes to measure the baseline signal *V*_0_. Subsequently, the target gases of predetermined concentrations are mixed and injected into the chamber. The infrared emitter is then activated and maintained for several minutes to ensure stable signal acquisition before being turned off. The tested concentration ranges for the target gases are 0–50,000 ppm for CO_2_, 0–25 ppm for CO, and 0–20,000 ppm for CH_4_.

To ensure accurate calibration and monitoring of the infrared source, a reference channel with a bandpass filter centered at 3.9 μm is integrated into the system. This filter was fabricated through a multilayer coating process to achieve high transmittance around 3.9 μm while blocking other wavelength regions, as shown by the yellow transmission peak in Fig. 3f. Since this wavelength is not absorbed by the target gases, the reference-channel signal mainly reflects the baseline intensity of the infrared beam and possible optical-path attenuation, rather than gas-specific absorption. Therefore, the 3.9 μm channel can be used to compare with the gas-responsive channels and compensate for drift in the emitter or sensor performance over time, improving the reliability of gas recognition. Upon activation of the infrared emitter, a corresponding increase in voltage is observed in each channel. After the emitter is turned off, the gas concentrations are adjusted, and the system is allowed to stabilize for a predefined interval before reactivating the emitter. This sequence is repeated to assess the BOD module’s response to various gas mixtures. The resulting relative voltage signals are presented in Fig. 5b, normalized by the baseline signal *V*_0_. Each curve shows the voltage signal from BOD, and the accompanying color bars indicate the concentrations of the respective target gases. Previous measurements demonstrated strong selectivity under interfering conditions. Thus, the observed voltage variations confirm that the BOD module can simultaneously detect multiple gases. The reference channel, centered at 3900 nm, remains stable throughout the measurements, indicating consistent infrared emission and enabling effective calibration against potential drift. As gas concentrations increase, the normalized voltage signals decrease due to enhanced infrared absorption at specific wavelengths. For CO_2_, the normalized voltage signal drops from 0.9 to 0.52 as the concentration increases from 0 to 50,000 ppm. Similarly, CO shows a decrease in normalized voltage from 0.82 to 0.36 over a concentration range of 0 to 25 ppm. CH_4_ exhibits a reduction in normalized voltage from 0.9 to 0.6 as its concentration increases from 0 to 20,000 ppm.

Notably, the BOD module demonstrates high sensitivity to CO at low concentrations, attributed to its higher effective absorption coefficient in the 3–5 µm spectral range compared to CO_2_ and CH_4_. The calibrated Beer-Lambert law curves were used to estimate gas concentrations from the measured voltage signals from each channel of BOD. Multiple measurements were conducted at each concentration, and the average values were used for estimation. Figure 5c summarizes the BOD response to ten different combinations of CO_2_, CO, and CH_4_ gas mixtures. In Fig. 5c, the upper left corner of each cell displays the set concentration values, while the lower right corner shows the corresponding measured values. The close color matching between the set and measured values indicates high measurement accuracy. The results reveal strong agreement at low concentrations, specifically, CO_2_ below 20,000 ppm, CH_4_ below 20,000 ppm, and CO below 10 ppm, highlighting the BOD module’s precision in this range.

Moreover, the scalability of the system arises from its modular architecture, whereby the incorporation of additional narrow-bandpass optical filters can extend the range of detectable gas species. The limit of detection (LOD) can be calculated based on the standard deviation *σ_y_* of the response curve and the slope *σ* of the calibration curve according to


(3) \begin{eqnarray*}
{\mathrm{LOD}} = 3 \times ({\sigma }_y/\sigma ).
\end{eqnarray*}

The device achieves remarkable detection limits, with a LOD of 1.42 ppm for CO_2_, 1.16 ppb for CO, and 0.15 ppm for CH_4_, demonstrating better performance compared to conventional TE-based NDIR gas sensors, which typically operate at the ppm level. The calculation data of LOD is shown in Fig. S11.

In addition, this sensing strategy principally can be extended to other gases that possess characteristic infrared-active vibrational absorption bands within the detectable spectral range. These IR absorption peaks originate from molecular vibrations accompanied by dipole-moment changes. Thus, polar or heteroatomic gas molecules generally exhibit characteristic absorption peaks in the infrared region, whereas non-polar gases such as H_2_, O_2_ and N_2_ are IR-inactive or show negligible absorption under the present detection mechanism. Besides CO_2_, CO, and CH_4_, representative examples include NH_3_, NO*_x_*, SO_2_, H_2_S, and various volatile organic compounds (VOCs) such as ethanol and acetone, all of which exhibit distinct infrared absorption features. By selecting appropriate narrow-band optical filters matched to the characteristic absorption wavelengths of these gases, the present platform could be further adapted for broader gas recognition. In contrast, gases with weak or negligible infrared absorption are difficult to distinguish using the current device configuration.

## CONCLUSION

We report the first fully integrated biomimetic olfactory device (BOD) based on an all-solid-state *i*-TE polymer array, which is fabricated using wafer-scale microfabrication processes. Using ions instead of electrons as charge and energy carriers, this BOD represents a significant advancement in gas sensing, addressing key challenges in power efficiency, sensitivity, and multi-gas detection. By adopting *i*-TE PVDF-HFP/NaTFSI/TPFPB/PC polymer with a large thermopower and a low thermal conductivity, the device achieves exceptional *S*/*κ* ratios of 24 mV·m/W, far surpassing the records of conventional electronic thermoelectric materials. As a result, this monolithic BOD achieves a responsivity of 2340 V/W, approximately 20 times higher than those of commercial thermoelectric detectors while operating without external power, driven solely by thermally induced cation/anion flux, which mimics the neural response initiation mechanism. The calculated LODs are 1.42 ppm, 0.15 ppm, and 1.165 ppb, demonstrating their great potential in applications requiring high sensitivity and selectivity. It should be noted that the present work focuses on three representative gases to validate the sensing concept. Nevertheless, the number and type of detectable gas species are primarily determined by the spectral selectivity of the optical filters and the array density, rather than by intrinsic limitations of the *i*-TE sensing mechanism. Clearly, these performance metrics, combined with the device’s scalability, environmental robustness, and low power consumption, position the BOD as a significant advancement for next-generation gas sensing across a wide range of industrial, environmental, and biomedical applications.

## MATERIALS AND METHODS

### Gas sensing measurement

A homemade measurement system has been developed for gas-sensing applications, incorporating three mass flow controllers (MFCs) with flow rates of 100, 200, and 500 standard cubic centimeters per minute (sccm). As the setup shown in Fig. 4a, MFC1 and MFC2 are designated for the injection of target gases, while MFC3 is utilized for delivering pure nitrogen as a carrier and diluting gas. The gases are mixed in a dedicated mixing chamber before being introduced into a custom-built testing chamber. The required flow rates for achieving these concentrations are calculated using the following equation:


(4) \begin{eqnarray*}
{V}_{{\textit {}T\!arget}} = \ ({C}_{\textit {}T\!arget\ PPM}/{C}_{{\textit {}Cylinder}\,PPM}) \times {V}_{{\textit {}T\!otal}}.
\end{eqnarray*}

*V_Target_* is the flow rate needed to achieve the desired concentration. *C_Target_  _PPM_* is the required concentration for the test. *C_Cylinder_  _PPM_* is the concentration of the gas from the supply cylinder. *V_Total_* denotes the total flow rate set on the mass flow controller. For the infrared light source, the commercial product EMIRS200 is employed, controlled via a Keithley 2400. The system operates with a voltage of 5.6 V and a current of 0.08 amperes for a duration of 10 minutes, followed by a reset to 0 V for another 10 minutes. For measuring voltage signals over time, a Keysight 34465A multimeter is integrated into the system, with control facilitated through a Java program. Additionally, three commercial products are utilized to monitor environmental parameters, including room temperature, humidity, and concentrations of CO_2_, CO, and CH_4_.

### Optical properties measurement of narrow-bandpass filters and an absorber

A Fourier transform infrared (FTIR) spectrometer (Vertex 70 Hyperion 1000) is employed to measure the transmission and reflection characteristics of the narrow bandpass filter and the absorption of the absorber. Calibration is performed using a gold reference. The absorption of the absorber is calculated from the measured reflection and transmission data using the following equation:


(5) \begin{eqnarray*}
a = 1-\rho -T,
\end{eqnarray*}

where *α* is the absorptance, *ρ* is the reflectance, and *T* is the transmittance.

### BOD sensing units process flow

The standard wafer cleaning process, known as RCA clean, is applied to the 4-inch, 500-micron-thick quartz wafers. A 300 nm thick layer of aluminum is deposited on the wafers using a sputtering process, with the power set to 100 W in DC mode. The deposition rate is approximately 40 nm/min, and the thickness of the aluminum is measured using a KLA-Tencor P-7 surface profiler. Next, the wafers are patterned using a Karl Suss MA6 aligner and etched with an Oxford aluminum etcher to create the bottom electrode (Fig. S12). A layer of SU-8 photoresist is then spin-coated onto the wafers, achieving a thickness ranging from 10 to 100 microns. Photolithography and development processes are employed to form a mold above the bottom electrode. The developed wafers are inspected under a microscope to ensure that the photoresist is fully developed and that the metal is exposed. To fill the SU-8 mold with ionic thermoelectric materials, a drop casting method is utilized (Fig. S13). The wafers are subsequently placed in a vacuum oven and baked at 60°C under a pressure of 10^−3^ Torr for 24 hours to evaporate most of the solvent, resulting in the formation of solid-state ionic thermoelectric pillars. A 300 nm thick layer of aluminum is then deposited on the wafers using a shadow mask in the sputtering process to create the top electrode (Fig. S14). The shadow mask is carefully aligned under a microscope to ensure proper coverage of the ionic thermoelectric pillars and the electrode. Finally, a layer of black paint is spray-coated onto the wafers using the shadow mask to form absorbers.

## Supplementary Material

nwag320_Supplemental_File
