# Supplementary material for: Low-power biomimetic ionic thermoelectric device for multi-gas olfaction
Source: Natl Sci Rev. 2026 May 28;13(11):nwag320. doi: 10.1093/nsr/nwag320 (PMC13289752; doi:10.1093/nsr/nwag320)
Supplement: nwag320_Supplemental_File [file nwag320_supplemental_file.pdf]

Supplementary Information

Low-power biomimetic ionic thermoelectric device for multi-gas olfaction

Gongze Liu[1,†], Cheng Chi[2,6,†,*], Jiacheng Ji[1,†], Keqiao Li[1], Meng Li[1], Yucheng Ding[3], Weiqi Zhang[3], Wenying Tang[3], Zhiyong Fan[3,*] and Baoling Huang[1,4,5,*]

[1]Department of Mechanical and Aerospace Engineering, The Hong Kong University of Science and Technology, Hong Kong, China;

[2]Key Laboratory of Power Station Energy Transfer Conversion and System of Ministry of Education, School of Energy Power and Mechanical Engineering, North China Electric Power University, Beijing 110026, China;

[3] Department of Electronic and Computer Engineering, The Hong Kong University of Science and Technology, Hong Kong, China;

[4] HKUST Shenzhen-Hong Kong Collaborative Innovation Research Institute Futian, Shenzhen 518000, China;

[5]Thrust of Sustainable Energy and Environment, The Hong Kong University of Science and Technology, Guangzhou 511400, China;

[6]School of Physics, Engineering and Computer Science, University of Hertfordshire, Hatfield AL10 9AB, United Kingdom

***Corresponding Authors.** chicheng@ncepu.edu.cn; eezfan@ust.hk; mebhuang@ust.hk

†Equally contributed to this work.

Materials

Polyvinylidene fluoride–hexafluoropropylene (PVDF-HFP) pellets (average molecular weight, 455,000 g mol^{-1}), sodium bis(trifluoromethylsulfonyl)imide (NaTFSI), propylene carbonate (PC, anhydrous, 99.7%), N-methyl-2-pyrrolidone (NMP; high-performance liquid chromatography, ⩾ 99%), and tris(pentafluorophenyl)borane (TPFPB, min. 97%) were purchased from Sigma-Aldrich. All the materials were stored in the glovebox without any additional treatment.

Preparation of *i*-TE material precursor solution

PVDF-HFP pellets were initially dried in an oven at 100°C for 24 hours and subsequently dissolved in NMP to achieve a concentration of 0.1 g mL^{-1}. The precursor solution was prepared by mixing 1 M NaTFSI with PC in a mass ratio of 86 weight percent, combined with the NMP solution containing the PVDF-HFP pellets, all within an argon atmosphere. This mixture was stirred vigorously using magnetic stirring for 8 hours. For the n-type material, the precursor solution was created by adding 0.25 M TPFPB to the precursor solution under the same argon atmosphere and stirred for an additional 8 hours at 60°C.

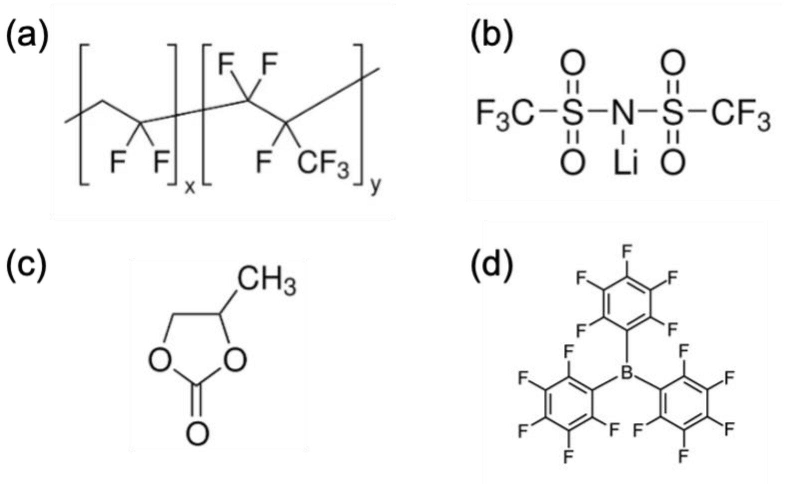

Fig. S1 The chemical structures of the molecules of the i-TE materials, including (a) PVDF-HFP, (b) NaTFSI, (c) PC and (d) TPFPB.

The PVDF-HFP serves as the ion-conducting polymer matrix, NaTFSI provides the mobile ions, and PC acts as the plasticizing solvent to facilitate ion dissociation and transport. After introducing TPFPB, the material is converted into an n-type ionic thermoelectric system.

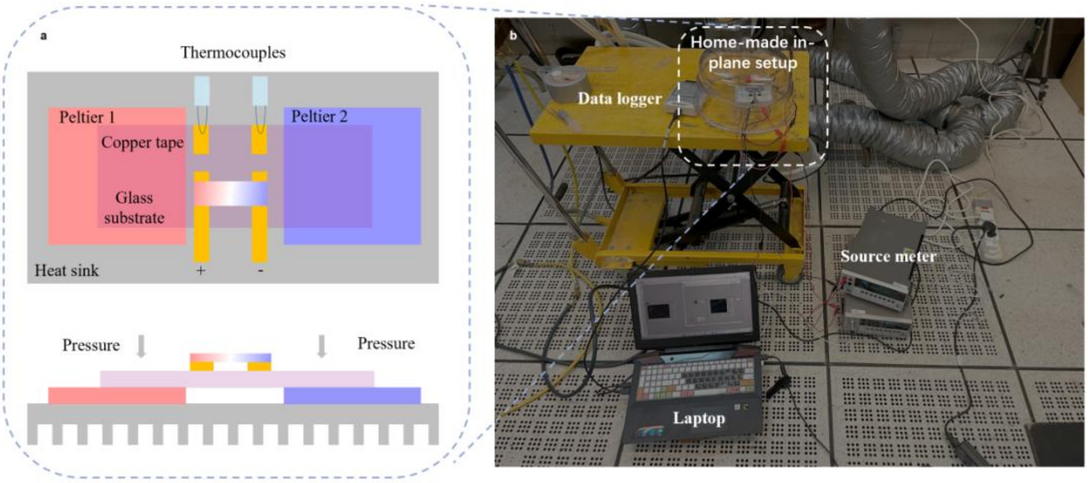

Fig. S2 Home-made in-plane setup. (a) Schematics of the setup (b) photograph of the setup.

The measurement platform for assessing the thermopower of *i*-TE materials consists of a homemade in-plane setup, a data logger, a source meter, and a laptop for data collection. This homemade setup incorporates thermocouples and copper tapes mounted on a glass substrate, along with two Peltier devices controlled by the source meter to establish a temperature gradient. A heatsink is also integrated into the system to enhance thermal management.

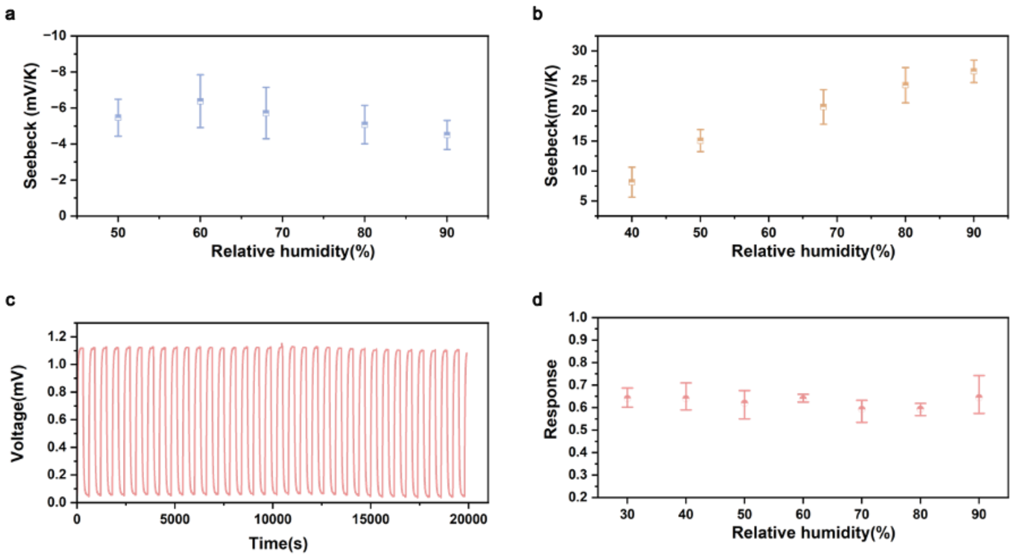

Fig. S3 Humidity measurement results. (a) Humidity response of n-type PVDF-HFP/NaTFSI/TPFPB/PC *i*-TE material and (b) the *i*-TE material PVDF- HFP/NaTFSI/ PC without TPFPB**.** (c) The response of BOD sensing unit to IR source (d) humidity response of BOD sensing unit.

As illustrated in Fig. S3, n-type PVDF- HFP/NaTFSI/TPFPB/PC *i*-TE material demonstrates better stability than that of without TPFPB under varying relative humidity conditions. Consequently, n-type *i*-TE materials were selected for the construction of BOD sensing units. These BOD sensing units underwent cycling under different relative humidity levels (Fig. S3c) and exhibited commendable stability, as shown in Fig. S3d.

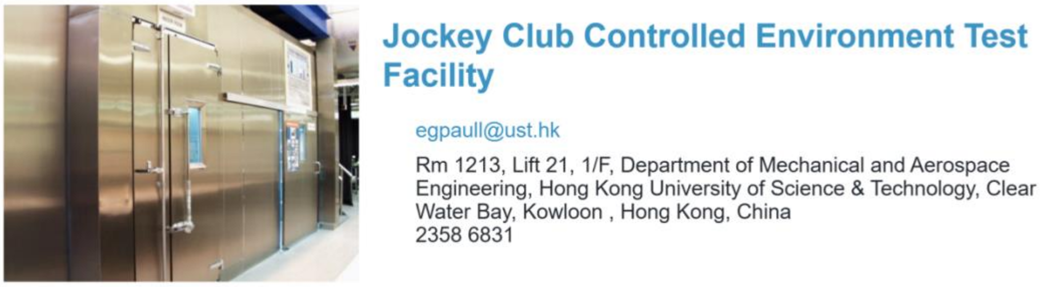

Fig. S4 Controlled environment test facility for relative humidity control and monitoring.

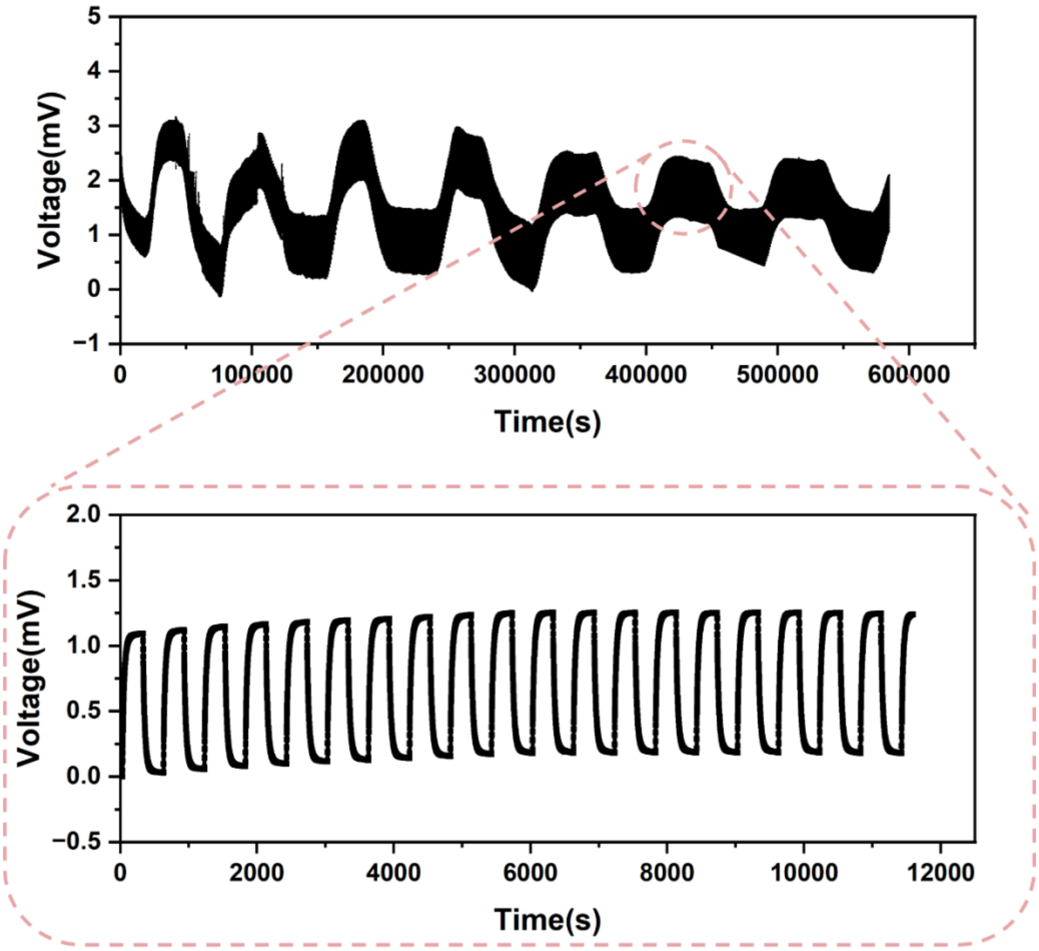

Fig. S5 Measurement results for 7 days.

The BOD was monitored over a period of 7 days, with the measured results presented here. Notably, during the measurement, the air conditioning was turned off at night and turned on during the daytime, leading to a periodic decrease and increase in the voltage signal. However, focusing solely on the voltage changes reveals that the voltage difference remains stable throughout the measurement period.

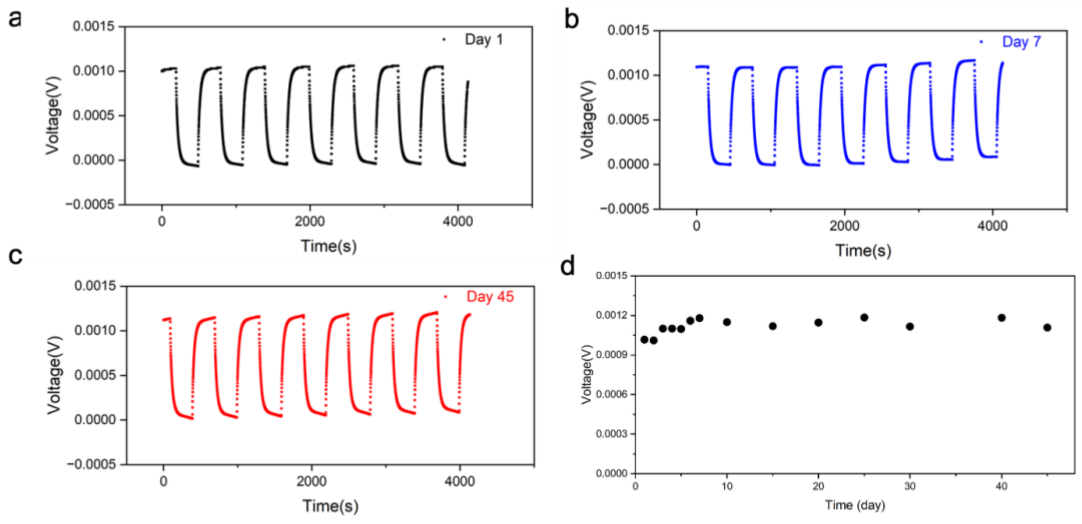

Fig. S6. The stability evaluation of the ionic thermoelectric BOD device. The representative output voltage responses of the BOD device measured at (a) day 1, (b) day 7 and (c) day 45. (d) The output voltage variations of the packaged BOD device monitored over 45 days under ambient conditions.

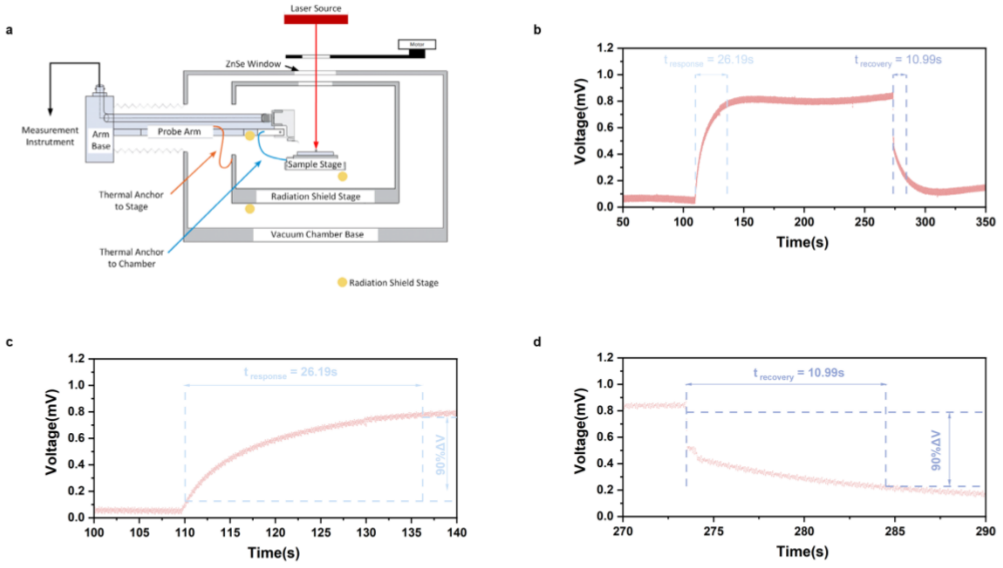

Fig. S7 Response time and recovery time measurements of BOD sensing unit. (a) The platform for response time measurement. (b) Response time values of BOD device. (c) Response time of BOD device. (d) Recovery time of BOD device.

Fig. S7 illustrates the response time measurement of the BOD sensing unit. Fig. S7a depicts the measurement platform, which comprises a laser light source, a chopper, a ZnSe window, and a probe station. Figs. S7b, c and d present the response time of the BOD sensing unit, demonstrating a response time of 26.19 seconds and a recovery time of 10.99 seconds.

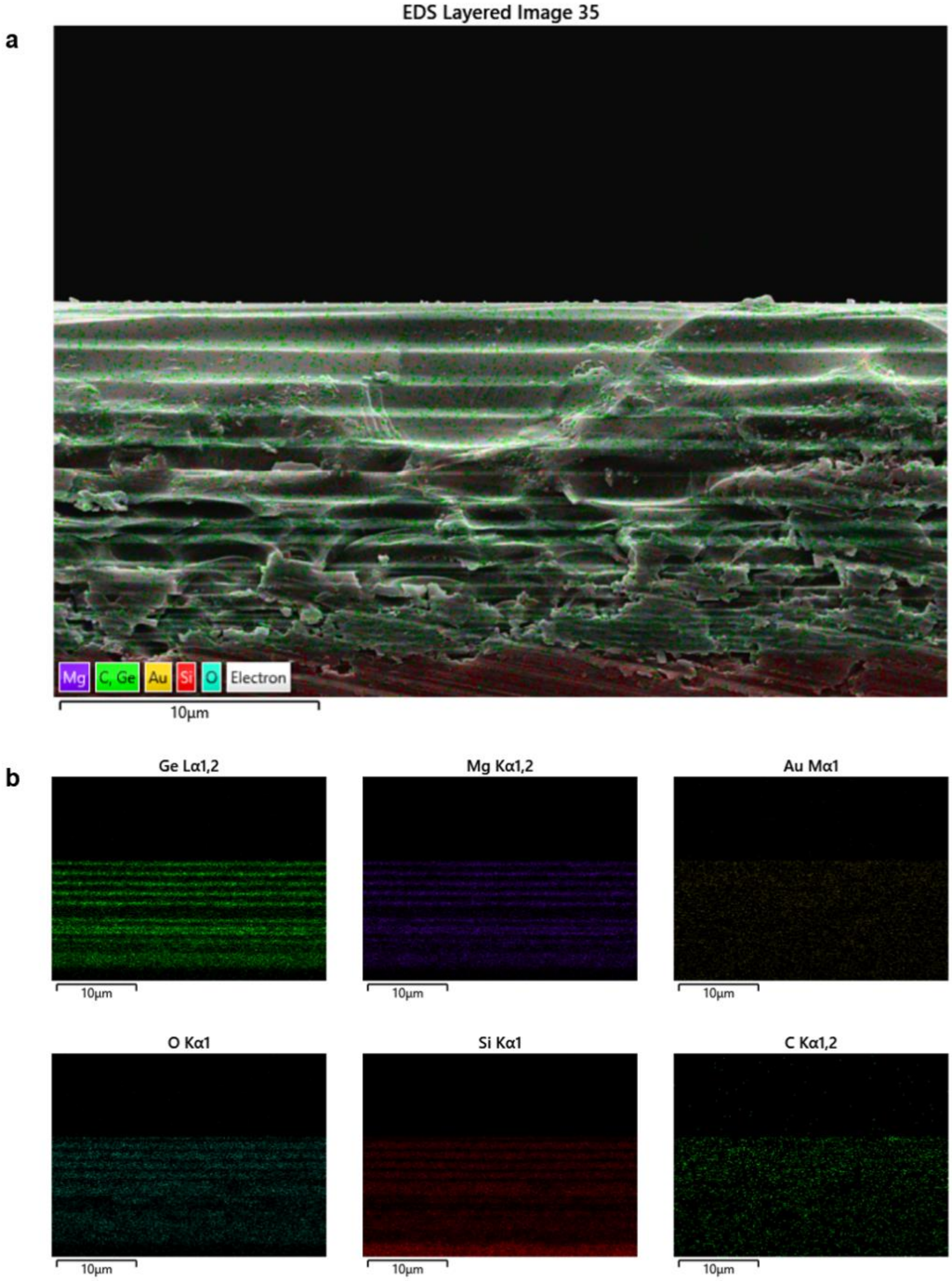

Fig. S8 Cross section of narrow bandpass filter. (a) SEM of the cross-section image (b) Element distribution of narrow bandpass filter.

Fig. S8 illustrates the cross section of narrow bandpass filter. Fig. S8a presents the optical profile of the filter, which consists of multilayer structures designed to enhance transmission at the desired spectral range. Fig. S8b displays the elemental distribution within this narrow bandpass filter.

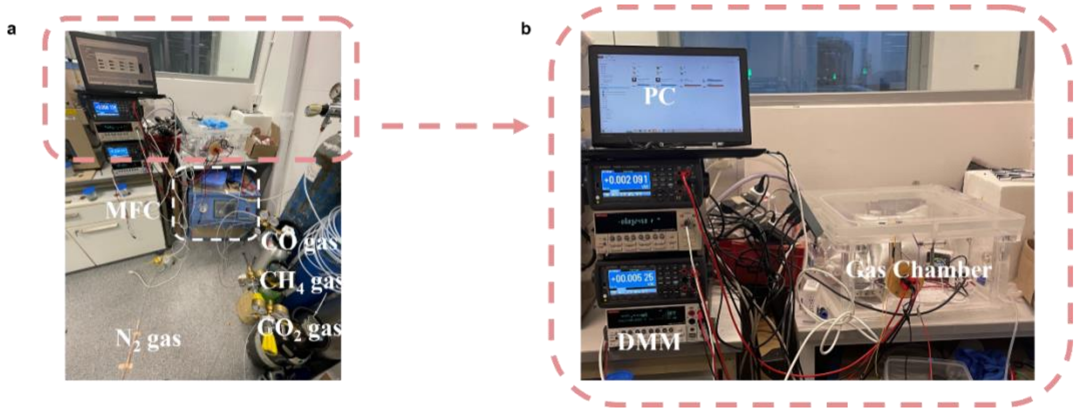

Fig. S9 The homemade measurement system. (a) Homemade measurement system. (b) Data collection system.

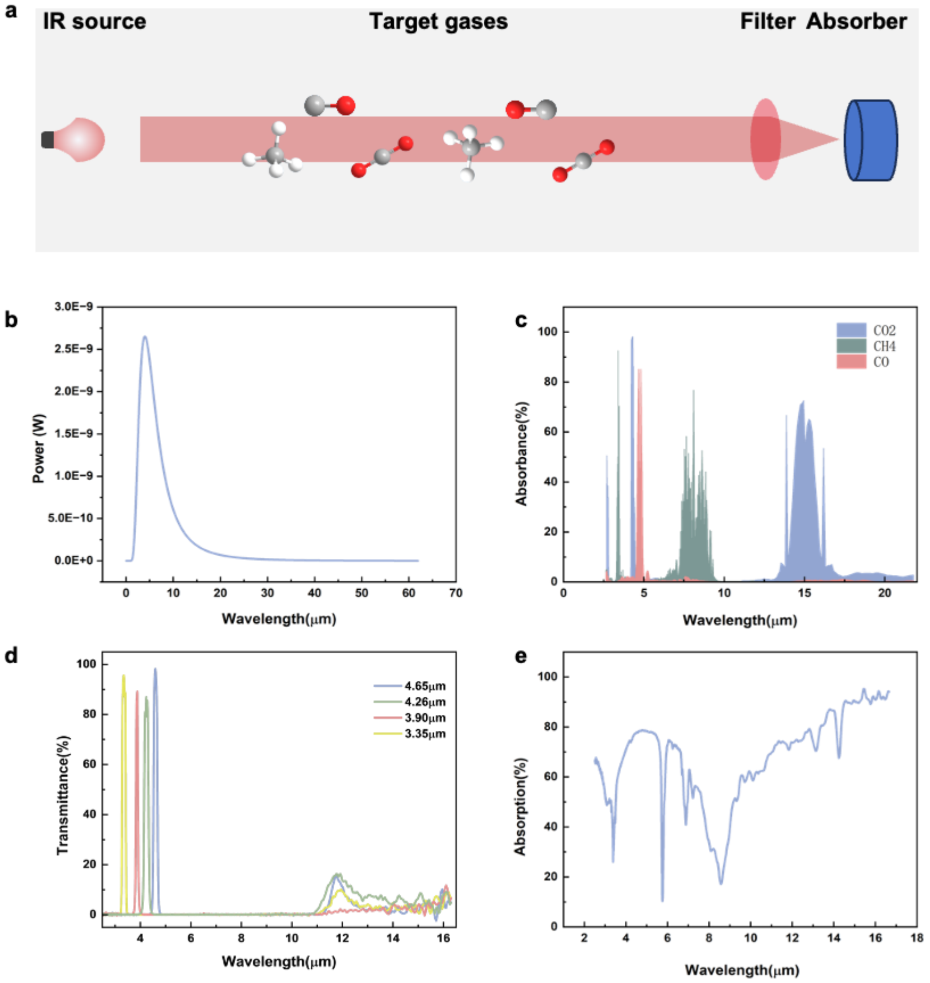

Fig. S10 The calculation of the effective absorption coefficient. (a) Schematic of the infrared transmission path. (b) Blackbody Spectra of EMIRS200. (c) Absorption spectra of the target gases, and (d) the measured transmittance spectra of the narrow bandpass filters and (e) measured absorption spectra of the absorber.

Fig. S10 presents the optical properties relevant to the calculation of the effective absorption coefficient. Fig. S10a shows the schematic of the infrared transmission path. Fig. S10b shows the Black Body Spectra of EMIRS200. Fig. S10c shows the absorption of the target gases extracted from the HITRAN database. Fig. S10d and S10e show the transmission of narrow bandpass filters and absorption of absorber, and the data is measured from FTIR.

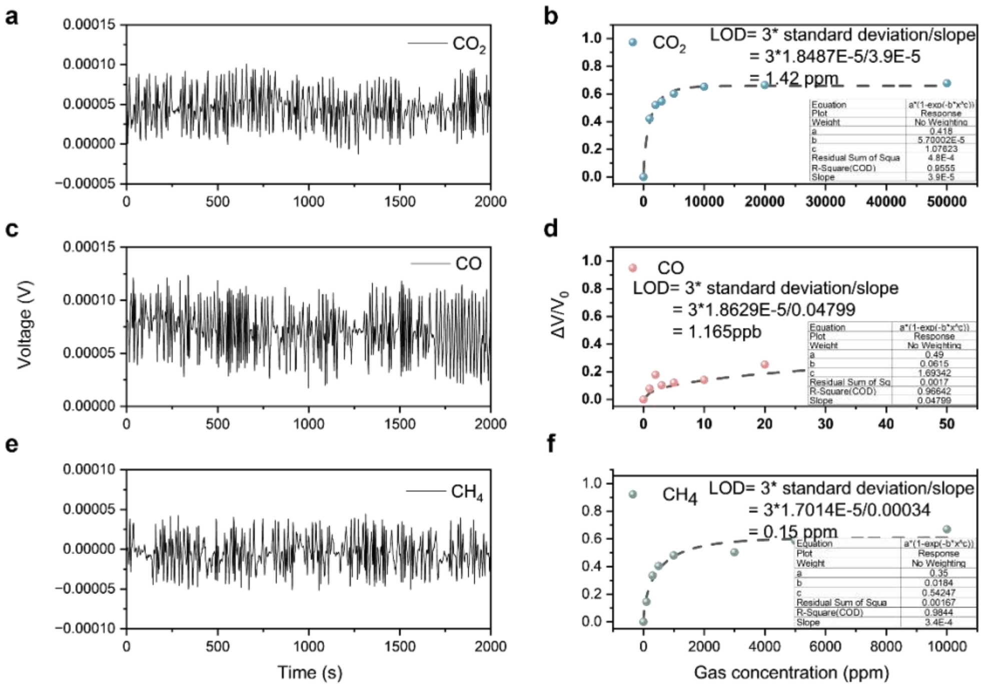

Fig. S11 Blank measurements and theoretical LOD estimations of the BOD. (a, c, e) Blank measurements for CO_2, CO and CH_4 channels, respectively. (b, d, f) Corresponding LOD estimations for CO_2, CO and CH_4, respectively.

Fig. S11 illustrates the theoretical limit of detection (LOD) results. The BOD was placed in gas chambers and supplied with pure wet nitrogen for 12 hours to establish a relatively stable baseline voltage signal. Following this, pure wet nitrogen was allowed to flow continuously through the chamber for 2000 seconds to collect data for calculating the relative standard deviation of the BOD. The slope used in this calculation was extracted from the fitted linear relationship presented in Fig. 4b.

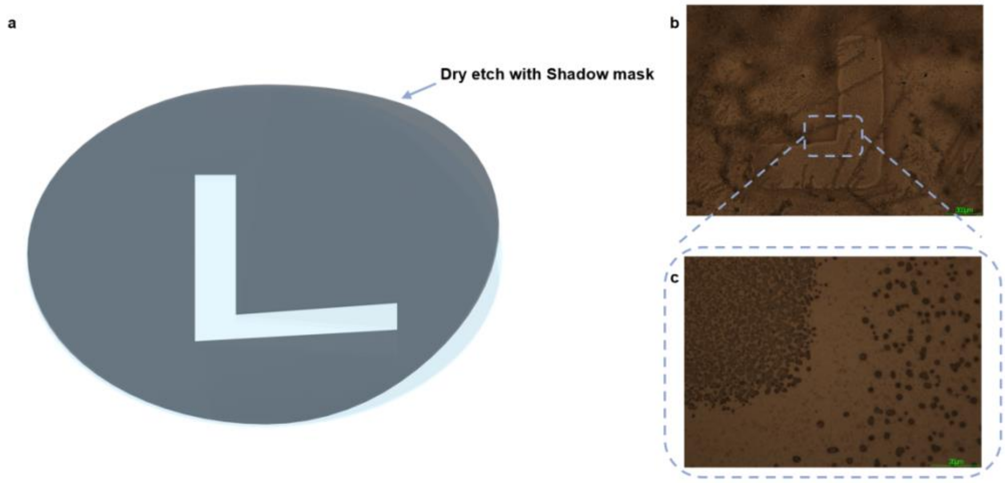

Fig. S12 Dry etch process for *i*-TE materials. (a) Shadow mask for dry etch (b) etching after 30 minutes (c) residues on glass wafer after dry etching.

The dry etching characteristics of *i*-TE materials were investigated using an L-shaped shadow mask alignment system, as demonstrated in Fig. S12. The substrate, consisting of spin-coated *i*-TE materials on glass, was subjected to oxygen plasma etching for 30 minutes through multiple cycles, with only the L-shaped regions exposed during the process. Microscopic analysis of the etched samples revealed significant challenges in achieving complete material removal. Substantial residues remained on the glass substrate following the etching procedure, which were identified as salt-based compounds through compositional analysis. These salt residues presented considerable obstacles to the etching process, effectively inhibiting further material removal and preventing complete pattern definition. The persistence of these residual compounds, combined with the difficulty in achieving their complete elimination through extended plasma exposure, suggests fundamental limitations in the dry etching approach for *i*-TE materials. The formation of non-volatile etch products appears to be intrinsic to the oxygen plasma interaction with the *i*-TE polymer matrix, leading to surface contamination that compromises both etch rate and selectivity. Based on these experimental observations, dry etching using oxygen plasma does not appear to be a viable processing method for *i*-TE materials patterning applications. The inability to achieve clean, residue-free pattern transfer indicates that alternative patterning strategies should be explored for successful integration of these materials into device fabrication workflows.

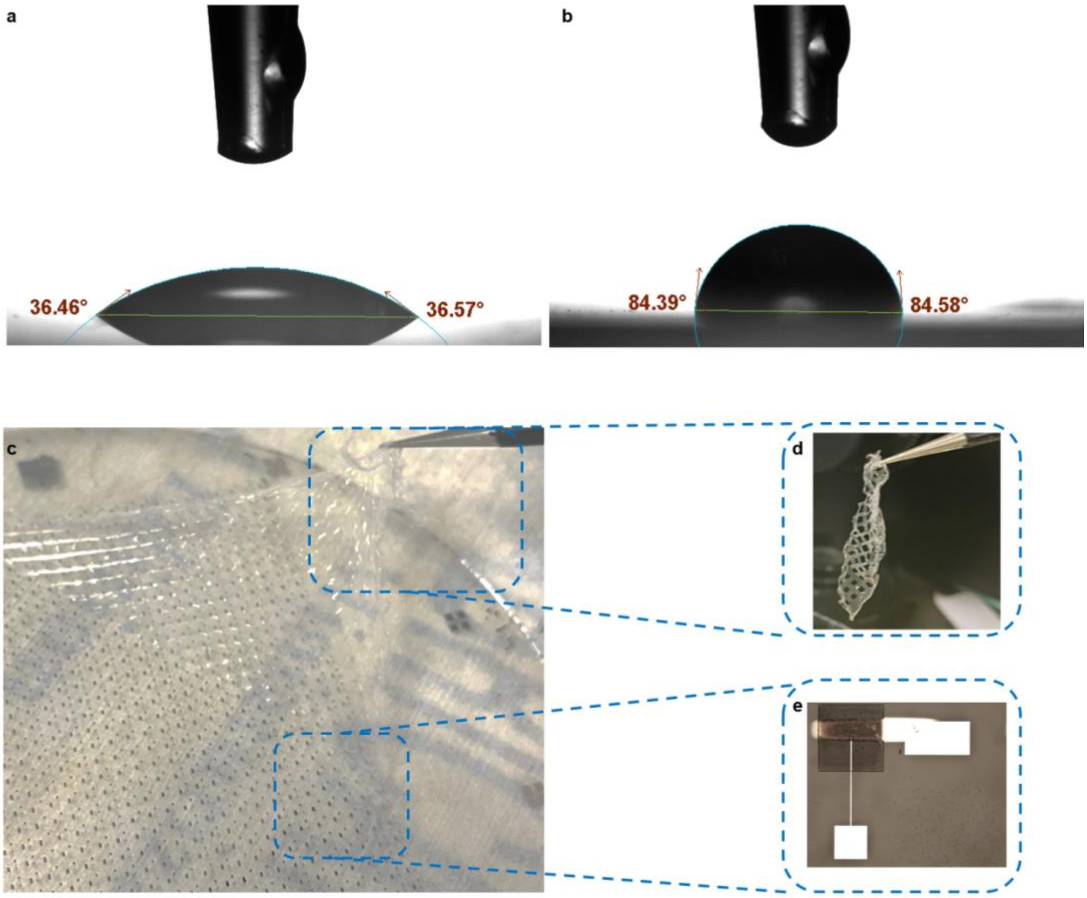

Fig. S13 *i*-TE material patterning. (a) Contact angle of n-type *i*-TE material on quartz wafer. (b) Contact angle of n-type *i*-TE material on SU-8. (c) Peeling process *i*-TE film. (d) Freestanding *i*-TE thin film after peeling (e) Patterned *i*-TE material.

Fig. S13 illustrates the patterning process of *i*-TE materials using an SU-8 mold. Contact angle measurements reveal a significant difference in surface interactions: the contact angle between the *i*-TE polymer and quartz is 36.46°, whereas it is 84.39° between the polymer and SU-8 (Figs S13a and S13b). This disparity in hydrophobicity facilitates the selective detachment of the *i*-TE polymer from the SU-8 mold after spin coating on a quartz wafer. Figures S13c to S13e demonstrate the overall patterning process and the integration of the patterned *i*-TE polymer into a BOD sensing unit.

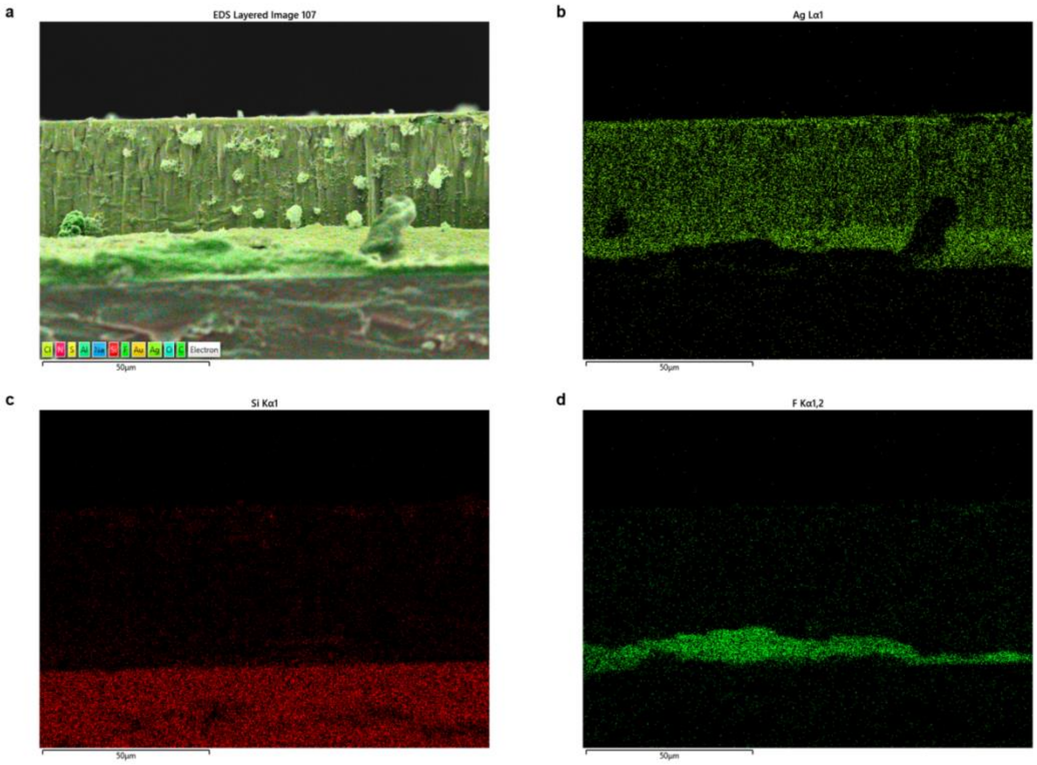

Fig. S14 Sidewall connection formed by sputtering process. (a) SEM of sidewall. (b) Ag distribution. (c) Si distribution. (d) F distribution.

Fig. S14 illustrates the sidewall connections formed through the sputtering process. Due to the difficulty in directly bonding the top surfaces with the CLCC ceramic package, a metal pad is pre-placed on the wafer. An aligned shadow mask is then used during the sputtering process to create the top electrode. This process inadvertently results in the formation of a sidewall that connects the top surface to the underlying metal pad. Fig. S14a to S14d show the element distribution of the sidewall, revealing that it is fully covered with metal following the sputtering process.

Supplementary Note 1

The effective absorption coefficient ε was calculated by the following equation with data from Fig. S10

$$\tau = \frac{I}{I_0} = \frac{\int_{\lambda_{min}}^{\lambda_{max}} S(\lambda)T(\lambda)A(\lambda)k(\lambda)d\lambda}{\int_{\lambda_{min}}^{\lambda_{max}} S(\lambda)T(\lambda)A(\lambda)\, d\lambda} = e^{-\varepsilon l_0 x_0}$$

$$\varepsilon = \frac{-\ln(\tau)}{l_0 x_0}$$

$S(\lambda)$ describes the spectral power of radiation from the IR source. A commercial product EMIRS200 is introduced, and the spectra follows the Planck radiation law. The spectral data could be found from https://www.heimannsensor.com/EMIRS-Series

$k(\lambda)$ represents the absorption of the target gases extracted from the HITRAN database www.spectraplot.com.

$T(\lambda)$ describes the transmittance of the narrow bandpass filters. the data is measured from Fourier-transform infrared spectroscopy (FTIR).

$A(\lambda)$ describes the absorption of absorber. the data is measured from FTIR.

The calculated value of k is shown below, the span and c values are fitted from the curves, and L values is measured from the gas chamber.

Table S1 The value of these parameters

Gas	Span	k(m^{-1})	c	L(m)
CO_2	6.589E-01	1.904E-04	7.327E-01	3.000E-01
CH_4	6.094E-01	6.195E-02	6.354E-01	3.000E-01
CO	8.000E-01	2.048E-01	4.851E-01	3.000E-01
